# Use of Slow Low‐Efficiency Dialysis in a Patient With Status Epilepticus Secondary to Herpes Simplex Encephalitis With Severe Acute Kidney Injury

**DOI:** 10.1155/crin/5586128

**Published:** 2026-09-27

**Authors:** Abigayle Therese R. Guiritan, Arlene C. Crisostomo

**Affiliations:** ^1^ St. Luke’s Medical Center, Quezon City, Philippines, stluke.com.ph

**Keywords:** acute kidney injury, herpes simplex encephalitis, SLED

## Abstract

**Introduction:**

Acute kidney injury (AKI) in the critical care unit is an independent risk factor for poor outcomes such as mortality. In patients with acute neurological injury and severe AKI, the timing and renal replacement therapy (RRT) modifications must be considered. Sustained low‐efficiency dialysis (SLED) is a cost‐effective alternative to continuous renal replacement therapy (CRRT) in critically ill AKI patients. We present a case of a 43‐year‐old male diagnosed with status epilepticus secondary to herpes simplex virus Type 1 (HSV‐1) central nervous system (CNS) infection who developed severe AKI and underwent SLED.

**Case Presentation:**

We present a case of a 43‐year‐old male, hypertensive, with no known chronic kidney disease (CKD) who was referred to nephrology service for management of AKI. He was being managed as a case of status epilepticus secondary to CNS infection. Diagnostic tests revealed elevated creatinine at 3.72 mg/dL from 0.9 mg/dL. Blood urea nitrogen was at 39 mg/dL. Sodium was at 148 mEq/L, and potassium at 3.4 mEq/L. Urinalysis had trace protein, moderate blood, and red blood cells at 12 cells per high power field (hpf). Creatine kinase MM (CK‐MM) was at 514 U/L (2.2x ULN). The patient also had marginal urine output at 0.2–0.4 mL/kg/h with the patient weighing 86 kg. Although the patient had Stage 3 AKI, RRT was initiated as a supportive measure. He underwent SLED for 6 hours with net ultrafiltration at 0 mL. Urine output gradually improved from 0.9 to 1.3 mL/kg/h. Repeat creatinine taken 2 days after showed a decreasing trend to 2.68 mg/dL. Seizures were controlled with midazolam drip, valproic acid, and lacosamide. Renal parameters were continuously monitored, and continuation of renal replacement was not deemed necessary. During his stay at the neurocritical care unit, lumbar puncture was performed, and cerebrospinal fluid tested positive for HSV‐1. Appropriate antimicrobials and methylprednisolone pulse therapy 1 g intravenously for 5 days were given. Neurologic status improved with a Glasgow coma scale of 11T (E4VTM6). Renal parameters were serially monitored, with repeat creatinine prior to discharge at 0.6 mg/dL.

**Conclusion:**

SLED can be used as a treatment modality in a neurocritically ill patient with status epilepticus for solute clearance. Good clinical judgment, early recognition, and ability to individualize care with management of AKI in the critical care unit can help improve clinical outcomes.

## 1. Introduction

Acute kidney injury (AKI) in the critical care unit has long been recognized as an independent risk factor for poor outcomes such as mortality [1–3]. In patients with acute neurological injury and severe AKI, the timing and renal replacement therapy (RRT) modifications must be considered because RRT can negatively impact cerebral blood flow, intracranial pressure, and brain tissue oxygenation, with effects tied to specific RRT methods [1]. Sustained low‐efficiency dialysis (SLED) is a cost‐effective alternative to continuous renal replacement therapy (CRRT) in critically ill patients with AKI as a mode of RRT [4]. We present a case of a 43‐year‐old male diagnosed with status epilepticus secondary to herpes simplex virus Type 1 (HSV‐1) CNS infection who developed severe AKI and underwent SLED.

## 2. Case Presentation

We present a case of a 43‐year‐old male, hypertensive, maintained on losartan 50 mg tab daily with blood pressure ranges of 120–130/80 mmHg, nondiabetic, with no known chronic kidney disease (CKD) who came in for continuity of care at our institution and was referred to the nephrology service for management of AKI. Few days prior, he developed intermittent fever accompanied by nonrotatory dizziness and one episode of vomiting after coming in from a local travel. He consulted at another hospital and was admitted and managed as a case of CNS infection. Initial cranial CT scan showed no evidence of acute intracranial hemorrhage, territorial infarct, focal edema, or discrete mass. He was started on ceftriaxone 2 g intravenously every 12 h, acyclovir 750 mg intravenously every 8 h, and mannitol 150 mL every 6 h. During the course of admission, he was persistently febrile with a maximum temperature of 38.5°C, with multiple generalized tonic–clonic seizures, with the longest lasting for more than 15 min, and was eventually intubated. On the third hospital day, relatives opted to transfer the patient to our institution, and he was admitted at the critical care unit with a Glasgow coma scale of 6T (E1VTM4). Cranial MRI was performed, which showed findings in the brain that were suggestive of encephalitic changes consistent with herpes encephalitis (Figure 1). Ceftriaxone was shifted to meropenem 2 g intravenously every 8 h, and vancomycin 1.3 g intravenously every 12 h was started. Acyclovir 750 mg intravenously every 8 h was continued. On the first hospital day, the patient had status epilepticus. Bolus doses of diazepam 5 mg intravenously were given, and valproic acid 1000 mg intravenously every 8 h was started. However, the seizure still persisted. The valproic acid level was subtherapeutic at 5.28 μg/mL, likely in the setting of concomitant meropenem therapy, which is known to cause a rapid and profound reduction in valproate concentrations through increased valproate clearance and reduced exposure. Lacosamide 200 mg twice a day and midazolam drip at 0.55 mg/kg/h were then started and titrated to maintain a Richmond Agitation‐Sedation Scale (RASS) of negative 4. Repeat laboratory tests were performed, which revealed creatinine elevation to 3.72 mg/dL from 0.9 mg/dL. Blood urea nitrogen was at 39 mg/dL. Sodium was at 148 mEq/L, and potassium at 3.4 mEq/L, which was corrected with free water flushing and potassium chloride drip and syrup via a nasogastric tube. Urinalysis had trace protein, moderate blood, and urine red blood cells at 12 cells per high power field (hpf). Creatine kinase MM (CK‐MM) was also elevated at 514 U/L, 2.2x the upper limit of normal, which could probably be secondary to the seizure episodes (Table 1). The patient also had marginal urine output at 0.2–0.4 mL/kg/h with the patient weighing 86 kg. Mean arterial pressure of ≥ 65 mmHg was maintained with norepinephrine drip, and hydration was continued with D5NM at 80 mL/hr. Although there was no absolute indication for RRT, in view of the patient’s concurrent renal and neurological deterioration, early initiation of SLED was pursued as an individualized kidney replacement strategy to facilitate gradual solute clearance while minimizing rapid osmotic and hemodynamic fluctuations. A central line was then inserted, and SLED with the following prescription was initiated: duration 6 h, blood flow (Qb) 150 mL/min, dialyzate flow (Qd) 300 mL/min, dialyzer F7HPS, heparin‐free, and bath: Na 140, K 4.5, Ca 3, HCO_3_ 35, with net ultrafiltration at 0 mL. After dialysis, urine output gradually improved from 0.9 to 1.3 mL/kg/h. Repeat creatinine taken 2 days after showed a decreasing trend to 2.68 mg/dL, and CK‐MM taken 3 days after was now within normal limits at 74 u/L. Seizures were eventually controlled with midazolam drip, valproic acid, and lacosamide. Renal parameters were continuously monitored, and continuation of renal replacement was not deemed necessary. During his stay at the neurocritical care unit, lumbar puncture was performed, and cerebrospinal fluid tested positive for HSV‐1. Appropriate antimicrobial was given for the infection, and methylprednisolone pulse therapy 1 g IV for 5 days was used as an adjunctive therapy to reduce CNS inflammation and edema. His neurologic status had gradual recovery with a Glasgow coma scale of 11T (E4VTM6), and he was able to communicate through gestures but had difficulty weaning from the ventilator. He eventually underwent tracheostomy and was weaned off from the ventilator, and the midazolam drip was discontinued. He was maintained on lacosamide 200 mg tablet and levetiracetam 1 g tablet twice daily. He was transferred to a regular room and was referred to rehabilitation medicine. Renal parameters were serially monitored, and creatinine eventually decreased to as low as 0.42 mg/dL, with repeat creatinine prior to discharge at 0.6 mg/dL (See Figure 2).

**FIGURE 1:**
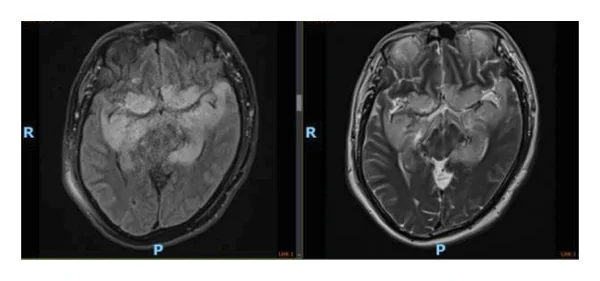
Cranial MRI suggestive of encephalitic changes consistent with herpes encephalitis.

**TABLE 1:** Summary of diagnostics at the time of acute kidney injury.

Complete blood count		Urinalysis		Renal parameters	
Hemoglobin	12 g/dL	Color	Light yellow	Creatinine	3.83 mg/dL
Hematocrit	35.6%	Transparency	Slightly hazy	BUN	36 mg/dL
Red blood cells (RBCs)	4.09 mil/mm^3^	Glucose	Negative	BUN creatinine ratio	9.4
White blood cells (WBCs)	11,590/mm^3^	Bile	Negative	Sodium	148 meq/L
Neutrophil	78%	Ketone	Negative	Potassium	3.9 meq/L
Lymphocyte	9%	Specific gravity	1.009	Chloride	114 meq/L
Eosinophil	5%	pH	5.5 (acidic)	Ionized calcium	1.17 mmol/L
Monocyte	8%	Protein	15 mg/dL (trace)	Bicarbonate	24 meq/L
Platelet count	1,75,000/mm^3^	Urobilinogen	Normal	Anion gap	10
MCV	87	Nitrites	Negative	Creatine kinase MM	514 U/L (NV: 35–232 U/L)
MCH	29	Blood	Moderate (2+)	Albumin	3.05 g/dL
MCHC	34	Leucocytes	Negative	Uric acid	5 mg/dL
RDW	12.8	Red blood cells	12/hpf	Arterial blood gas	
Therapeutic drug monitoring	White blood cell	2/hpf	pH	7.46	
Valproic acid	5.28 μg/mL (L) NV: 50–100	Epithelial cell	1/hpf	pCO_2_	33
Vancomycin trough level	31.79 μg/mL (H)	Cast	2/hpf	paO_2_	76
Infectious workup	Bacteria	46/hpf	Bicarbonate	23.5	
Meningitis encephalitis panel	HSV‐1 detected	RBC information	isomorphic	O2 saturation	97.6% at AC mode FIO_2_ 30%

**FIGURE 2:**
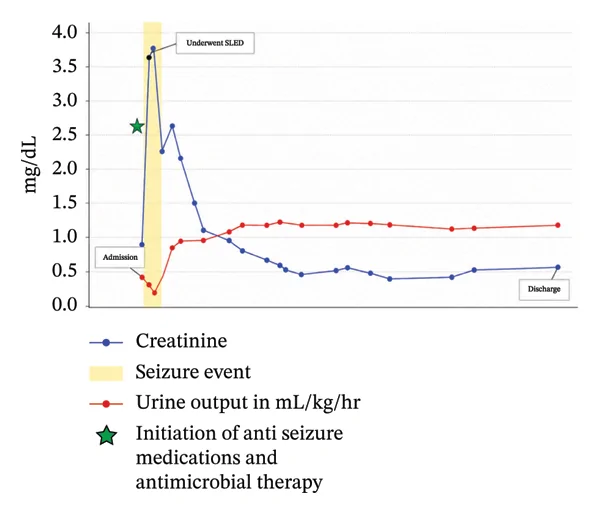
Summary of key events of the patient during the course of admission.

## 3. Discussion

HSV‐1 is the most common etiology of sporadic encephalitis worldwide and is associated with significant morbidity and mortality in adults and children despite antiviral treatment [5]. A lot of factors may have contributed to the patient’s AKI such as the use of nephrotoxic agents like vancomycin and acyclovir and probable rhabdomyolysis from status epilepticus. The patient was initially started on vancomycin 1.3 g intravenously every 12 h, with repeat vancomycin trough level being supratherapeutic at 31.79 μg/mL, coinciding with marginal urine output and rising creatinine. The mechanism of vancomycin‐associated AKI is not yet fully understood, but main mechanisms include high intracellular drug concentration in the renal tubules of individuals with risk factors for nephrotoxicity, subsequently inducing oxidative stress, complement activation, inflammatory injury, mitochondrial dysfunction, and cellular apoptosis in proximal renal tubules [6]. Vancomycin was put on hold, and hydration with D5NM was maintained at 80 mL/hr. Another factor that may have predisposed the patient to AKI was the use of acyclovir. However, the patient’s dose was at 750 mg IV q8 (8.7 mg/kg) as treatment for HSV encephalitis, which was adjusted based on creatinine clearance. Both acyclovir and vancomycin are renally cleared and may contribute to the development of AKI. Status epilepticus is an important nontraumatic cause for rhabdomyolysis. Urinalysis showed microscopic hematuria, but CK‐MM in our patient was only mildly elevated at 514 U/L and less likely to be the main cause of AKI. Based on the history, physical examination, and diagnostic examinations, he had Stage 3 AKI, which was defined as a 3‐fold increase of serum creatinine from the baseline [7]. This is most probably secondary to an intrinsic cause from the use of nephrotoxic agents. Vancomycin was promptly discontinued, acyclovir was revised to 8.7 mg/kg every 24 h (SLED dosing) to account for the patient’s severe renal impairment and the altered drug clearance associated with kidney replacement therapy, and hydration was at 80 mL/hour to target a fluid balance of positive 500 mL per day. A potential confounder for the patient’s seizure could have been the meropenem–valproate interaction. Combining valproate derivatives with carbapenem antibiotics is associated with a potential drug interaction that decreases serum concentration of valproate and may expose the patient to uncontrolled seizure risk from subtherapeutic valproate concentration [8]. In a patient with encephalitis who is actively having a seizure with increasing creatinine trends, early dialysis was performed instead of being conservative about the indications because developing uremia can worsen brain dysfunction. Our patient was diagnosed with encephalitis, and development of uremia can compound neurologic injury, lowering the seizure threshold and worsening the encephalopathy. A decision was made to use SLED as an individualized kidney replacement strategy to facilitate gradual solute clearance while minimizing rapid osmotic and hemodynamic fluctuations, thereby offering potential advantages in patients with severe AKI and concomitant acute neurological injury. SLED is a prolonged intermittent kidney replacement therapy (PIKRT), also referred to as prolonged intermittent renal replacement therapy (PIRRT), with similar short‐term outcomes to CRRT, including mortality and renal recovery, while providing more rapid solute clearance than CRRT [9]. In a study by Sharieff et al., they found that SLED can be considered as an alternative to CRRT for selected hemodynamically unstable patients requiring RRT [10]. SLED was performed instead of conventional hemodialysis since our patient is on one vasopressor support and has encephalitis, which can increase the risk of cerebral edema and dialysis disequilibrium. This renal replacement modality can minimize osmotic shifts, which can be similar to physiologic benefits observed with CRRT. Our patient completed the SLED with no intradialytic events noted.

## 4. Conclusion

SLED can be used as a treatment modality in a neurocritically ill patient with status epilepticus for solute clearance. Good clinical judgment, early recognition, and ability to individualize care with management of AKI in the critical care unit can help improve clinical outcomes.

## Funding

This study received partial financial assistance from the Section of Nephrology, St. Luke’s Medical Center‐Quezon City, Philippines, for the article processing charge.

## Consent

Written informed consent for publication and personal data processing was obtained from the patient or, when the patient was unable to provide consent, from their relative or legal guardian.

## Conflicts of Interest

The authors declare no conflicts of interest.

## Data Availability

The data that support the findings of this study are available on request from the corresponding author. The data are not publicly available due to privacy or ethical restrictions.
